# Supplementary material for: Acrylamide Hydrogel-Modified Silicon Nanowire Field-Effect Transistors for pH Sensing
Source: Nanomaterials (Basel). 2022 Jun 16;12(12):2070. doi: 10.3390/nano12122070 (PMC9227456; doi:10.3390/nano12122070)
Supplement: Supplementary file 1 [file nanomaterials-12-02070-s001.zip › nanomaterials-1686527-supplementary.pdf]

Supporting Information

Acrylamide Hydrogel-Modified Silicon Nanowire Field-Effect Transistors for pH Sensing

Gangrong Li [1,2,3,†], Qianhui Wei [1,2,†,*], Shuhua Wei [4], Jing Zhang [4], Qingxi Jin [1,2,3], Guozhi Wang [1,2,3], Jiawei Hu [4],
Yan Zhu [1], Yun Kong [1], Qingzhu Zhang [5], Hongbin Zhao [1,2], Feng Wei [1,*] and Hailing Tu [1,2]

1 State Key Laboratory of Advanced Materials for Smart Sensing, GRINM Group Co., Ltd., Beijing 100088, China; gangrongli@foxmail.com(G.L.); jinqingxi@foxmail.com(Q.J.); wangguozhi0809@126.com (G.W.); zhuyancxy@foxmail.com (Y.Z.); yunkong0503@163.com (Y.K.); zhaohongbin@grinm.com (H.Z.); tuhl@grinm.com (H.T.)
2 GRIMAT Engineering Institute Co., Ltd., Beijing 101407, China
3 General Research Institute for Nonferrous Metals, Beijing 100088, China
4 School of Information Science and Technology, North China University of Technology, Beijing 100144, China; weishuhua@ncut.edu.cn (S.W.); zhangj@ncut.edu.cn (J.Z.); hujiawei@ime.ac.cn (J.H.)
5 Advanced Integrated Circuits R&D Center, Institute of Microelectronic of the Chinese Academy of Sciences, Beijing 100029, China; zhangqingzhu@ime.ac.cn
* Correspondence: weiqianhui@grinm.com (Q.W.); weifeng@grinm.com (F.W.)
† These authors contribute equally to this work.

Preparation of Buffer Solutions of Varying pH

Preparation of ion concentration 0.01 mol/L phosphate buffer (1×PBS): weigh out 0.14196 g Na_2HPO_4 and 0.11998 NaH_2PO_4 and dissolve in 100 mL deionized water. Fully dissolve under the ultrasonic of an ultrasonic cleaner, and then take appropriate amount of NaH_2PO_4 solution and Na_2HPO_4 solution, as well as HCL, NaOH solution, adjust the pH value through a pH meter to prepare solutions with pH 3, 5, 7, 9, 11 and 13.

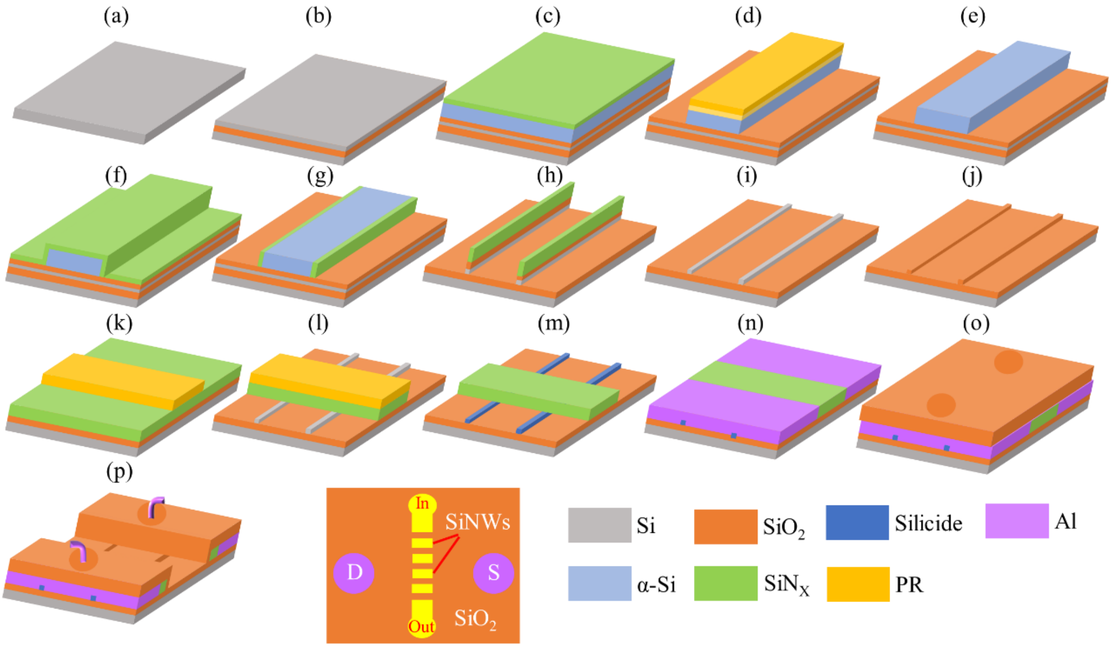

Figure S1. Fabrication flow of SiNW-FET

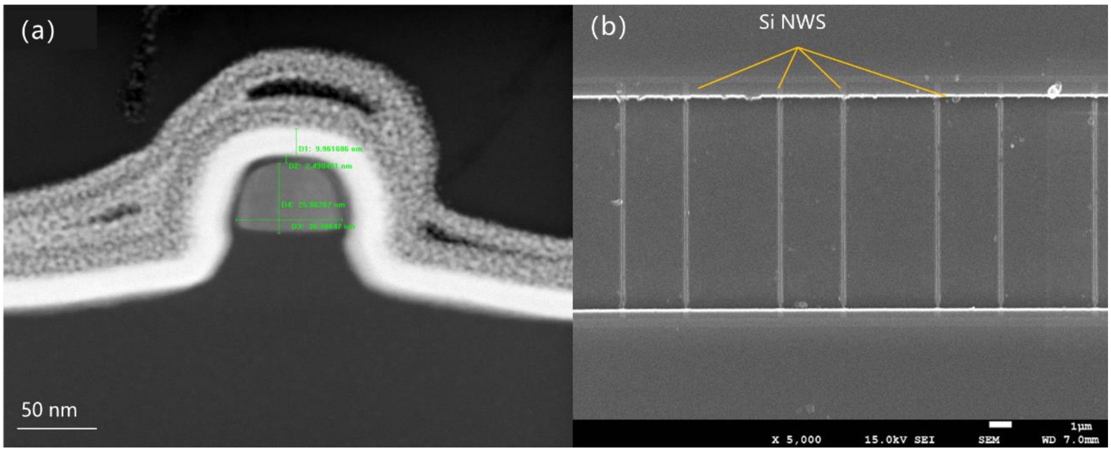

Figure S2. (a)TEM image of Si NW channel of the device, (b) SEM image of Si NW array in top view.

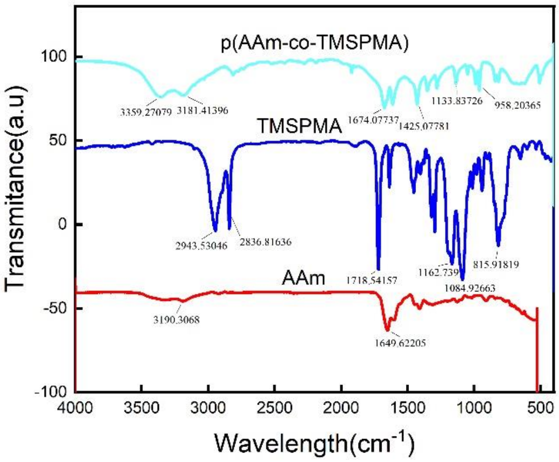

Figure S3. FTIR measurement of (red line) acrylamide, (blue line) 3-mercaptopropyl-trimethylsilane and (green line) hydrogel-modified silicon wafer.

As depicted in Figure S2, the two infrared characteristic peaks of AAm indicate that the amide group, 3190 cm^{-1}, is the stretching vibration absorption peak of -NH of the primary amine in the amide, and there is a -C=O stretching vibration absorption peak at 1649 cm^{-1}. For TMSPMA, 2841 cm^{-1} is the stretching vibration absorption peak of -CH, and there is a -C=O stretching vibration absorption peak at 1720 cm^{-1}. The 1162 cm^{-1} corresponds to the stretching vibration of the C-O alkoxy group, and the absorption peaks at 1087 cm^{-1} and 815 cm^{-1} are the asymmetric stretching vibration and the symmetrical stretching vibration of the Si-OH bond, respectively. During the configuration process of the solution, since the addition of TMSPMA will react with AAm and other TMSPMA, the absorption peak of Si-O bond in the infrared spectrum of the nanogel is weakened, and the position of Si-O-Si bond is analyzed. 3357 cm^{-1} is the stretching vibration absorption peak of -NH of the primary amine in the amide, and there is a -C=O stretching vibration absorption peak at 1669 cm^{-1}. 3181 cm^{-1} is the stretching vibration absorption peak of -CH, and there is a strong absorption peak at 1422 cm^{-1}, which should be the characteristic peak of stretching vibration absorption of C-O-C in the ester group.

To obtain hydrogel swollen structure, we design a strategy to preparation of powder products by freeze drying. Typically, the hydrogel paint is prepared according to experimental section. The as-prepared hydrogel paint is subject to freezer at -80 C overnight. The resulting frozen hydrogel paint is put in lyophilizer, which is set at -20 C at high vacuum for 2 days to remove water. The hydrogel solution becomes white porous solid, as depicted in Figure S4.

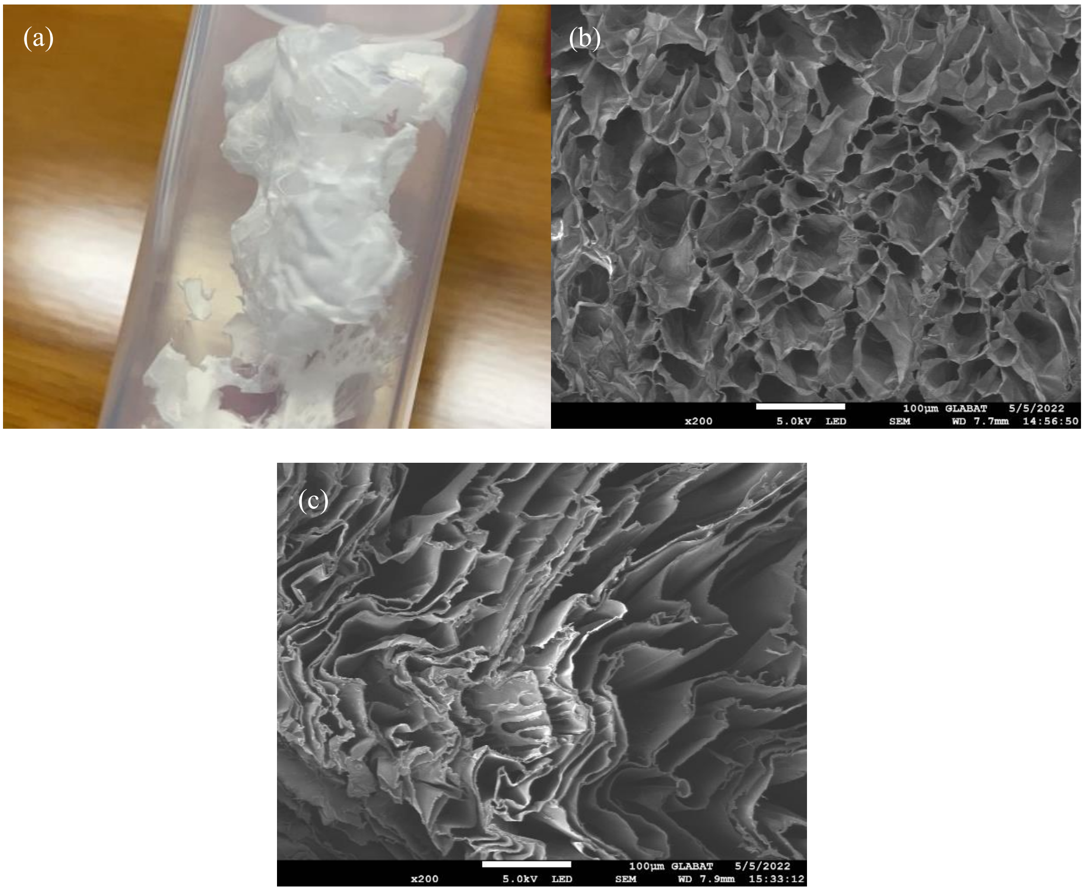

Figure S4. (a) Image of the white porous solid after freeze-drying of the hydrogel solution. (b) SEM image of freeze-drying of the hydrogel solution in pH = 3, (c) SEM image of freeze-drying of the hydrogel solution in pH = 7.

We found that the hydrogel exhibited a point-like porous structure when the pH value is 3. While the hydrogel exhibited a lamellar porous structure when pH value is 7 or 13, greatly increasimg the swollen volume of the hydrogel and the size of the mesh size. It would increase the diffusion of solution ions.

The I_D-V_G and I_D-V_D curves by bias gate voltages of the SiNW FET sensors are shown in Figure S5. The electrical characteristic curve exhibits typical p-type field effect behavior. For the electrical characteristic curve of V_D=2 V, the calculated switching ratio and extracted values of SS is about $4.81×10^5$ and 1.75 V/dec, respectively. When V_G increases from -20 to 0 V, the electrical signal response of the device decreases, showing the characteristics of a pMOS field effect transistor. The drain current increases with increasing VG bias, implying that the carrier concentration inside SiNWs can be linearly adjusted, and devices exhibit good FET electrical performance.

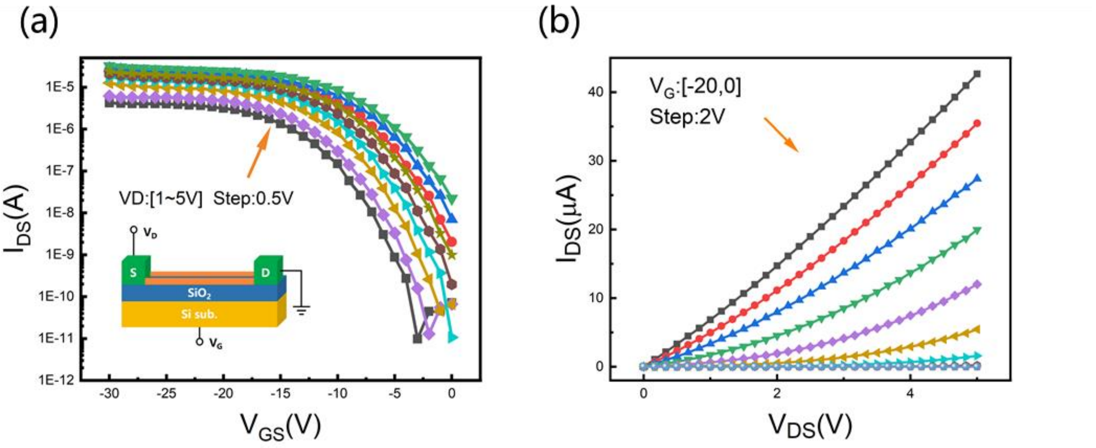

Figure S5. Electrical characteristic curve of SiNW-FET, (a) Transfer characteristic curve (I_{DS}-V_{GS}), the inset is a schematic diagram of device detection. (b) Output characteristic curve (I_{DS}-V_{DS}).

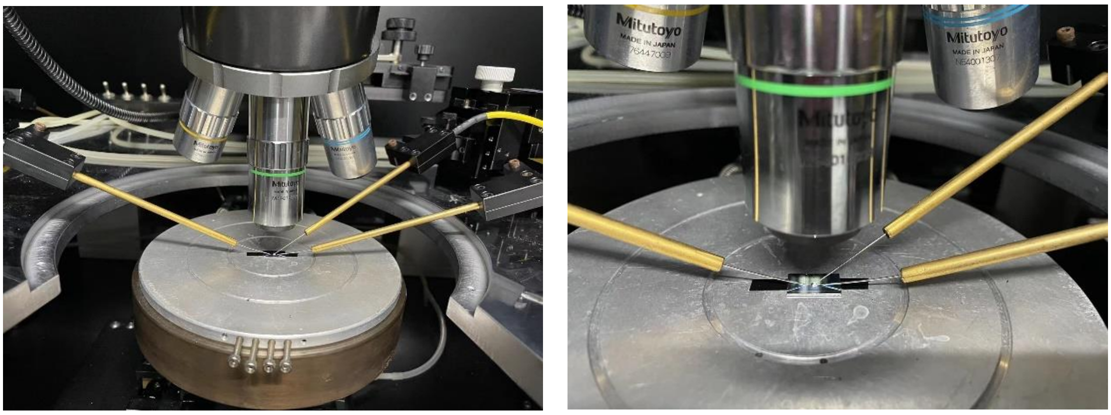

Figure S6. The actual test environment using the Keithley 4200A-SCS semiconductor parameter analyzer.

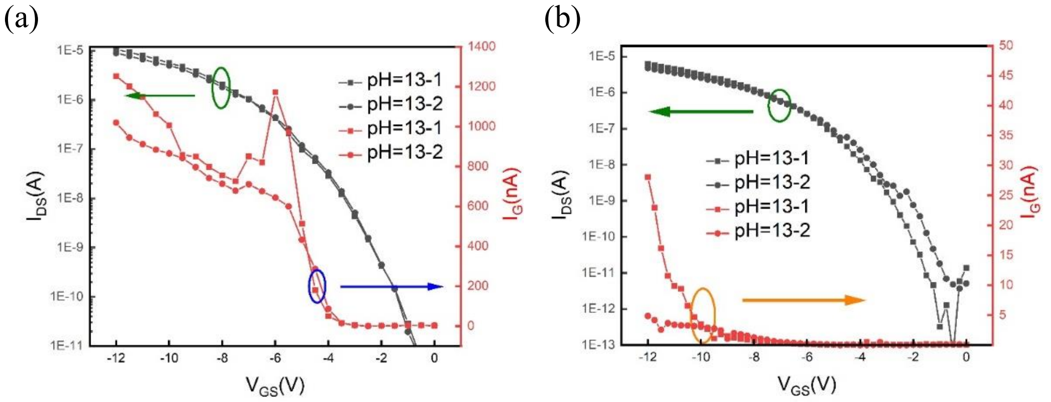

Figure S7. (a) Electrical characteristic curve of (I_{DS}-V_{GS}) and (I_G-V_{GS}) for SiNW-FET before modification, (b) Electrical characteristic curve of (I_{DS}-V_{GS}) and (I_G-V_{GS}) for hydrogel functionalized SiNW-FET.

EIS spectra (in terms of real and imaginary parts of the overall complex impedance Z measured

at the gate/electrolyte interface, Figures S8b and S8c) were acquired as a function of the angular frequency $\omega = 2\pi f$ (with f varying in the range between 0.1 and 100 kHz, for a perturbation amplitude of 10 mV). Fitting such measured impedance using the equivalent circuit shown in Figure S8e allows the estimation of the value of the capacitance in dc conditions needed for transistors characterization, namely Cgate/electrolyte. Impedance spectra can be also used to determine the capacitance dependence on frequency. Indeed, under the hypothesis of a totally blocking electrode (i.e., an electrode where no faradaic reactions occur) the imaginary part Z_{im} of the total impedance is almost purely capacitive, and as a consequence, capacitance can be expressed according to the following formula: $C = -1/(\omega Z_{im})$ (Figure S8f). Capacitance values at very low frequencies are consistent with the dc values extracted from the overall impedance spectra fitting.

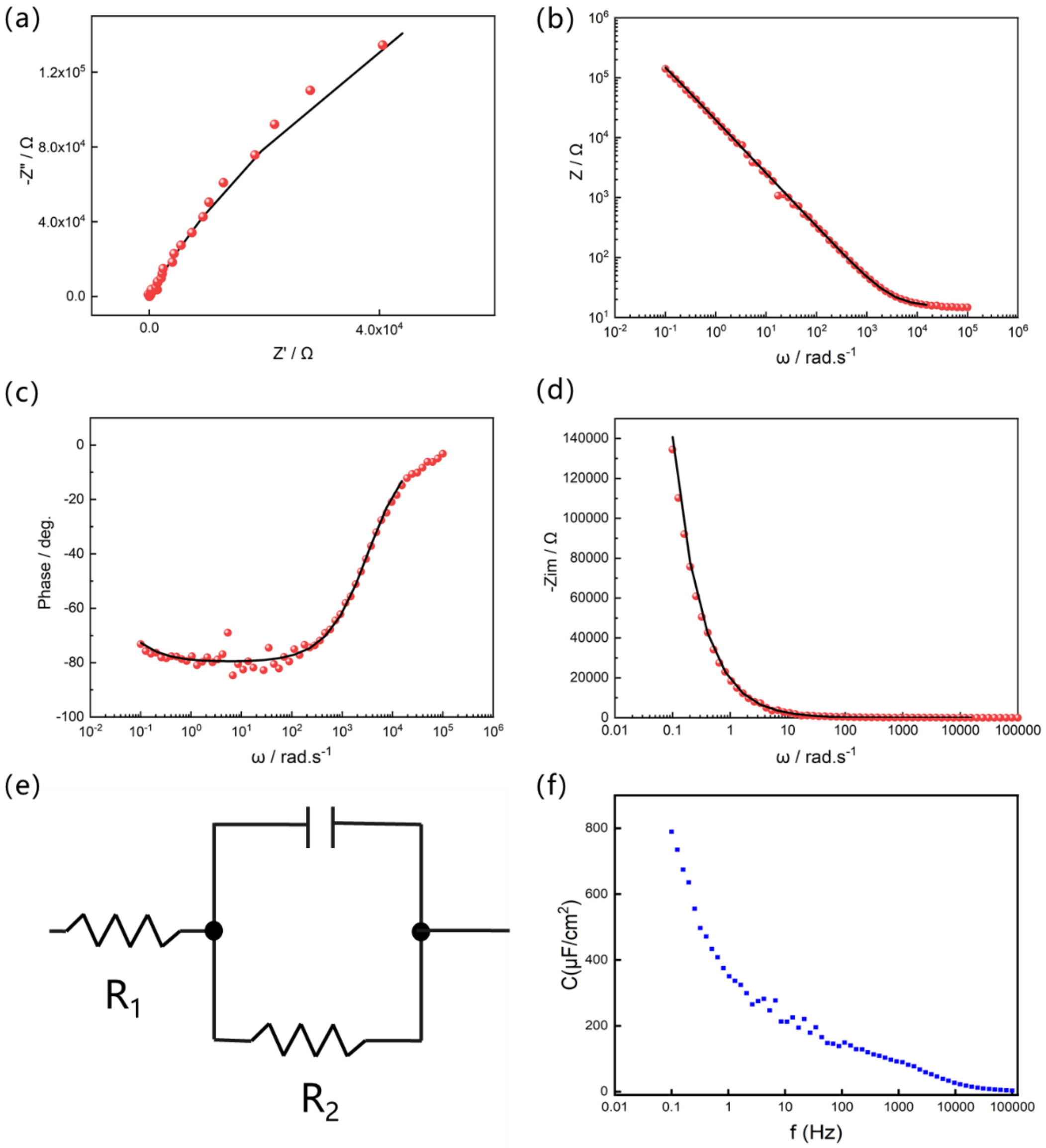

Figure S8. (a) Nyquist, (b) Bode, (c) phase diagram and (d) reactance of hydrogel-modified gate (pH=13). (●) Experimental data and (black line) fitting results to experimental data using the equivalent circuit shown in (e). (f) Capacitance obtained from $C=-1/(2\pi f Z_{im})$
